# Zero-Shot Artifact2Artifact: Self-incentive artifact removal for photoacoustic imaging

**DOI:** 10.1016/j.pacs.2025.100723

**Published:** 2025-04-18

**Authors:** Shuang Li, Qian Chen, Chulhong Kim, Seongwook Choi, Yibing Wang, Yu Zhang, Changhui Li

**Affiliations:** a College of Future Technology, Peking University, Beijing, China; b Department of Electrical Engineering, Convergence IT Engineering, Mechanical Engineering, and Medical Science and Engineering, Medical Device Innovation Center, Pohang University of Science and Technology (POSTECH), Pohang, Republic of Korea; c National Biomedical Imaging Center, Peking University, Beijing, China

**Keywords:** 3D photoacoustic imaging, Artifact removal, Zero-shot, Sparse detector array

## Abstract

Three-dimensional (3D) photoacoustic imaging (PAI) with detector arrays has shown superior imaging capabilities in biomedical applications. However, the quality of 3D PAI is often degraded due to reconstruction artifacts caused by sparse detectors. Existing iterative or deep learning-based methods are either time-consuming or require large training datasets, limiting their practical application. Here, we propose Zero-Shot Artifact2Artifact (ZS-A2A), a zero-shot self-supervised artifact removal method based on a super-lightweight network, which leverages the fact that patterns of artifacts are more sensitive to sensor data loss. By randomly dropping acquired PA data, it spontaneously generates subset data to reconstruct images, which in turn stimulates the network to learn the artifact patterns in reconstruction results, thus enabling zero-shot artifact removal. This approach requires neither training data nor prior knowledge of the artifacts, making it suitable for artifact removal for arbitrary detector array configurations. We validated ZS-A2A in both simulation study and invivo animal experiments. Results demonstrate that ZS-A2A achieves high performance compared to existing zero-shot methods.

## 1 Introduction

As a promising imaging modality, photoacoustic imaging (PAI) combines ultrasound detection with optical absorption contrast to image deep in living tissue with high spatial resolution [1], [2], [3], [4], [5], [6]. Furthermore, 3D PAI systems with spherical or planar array configurations have gained much attention and made rapid progresses [7], [8], [9], [10], [11], [12], [13], [14]. However, due to cost and technical difficulties, most real-time 3D PAI systems only have sparse and angle-limited sensor arrays in reality, which desires advanced algorithms to reduce artifacts and enhance image quality of PAI results reconstructed with fast traditional back-projection (BP) algorithms, such as delay and sum (DAS) and universal filtered back-projection (UBP). To address this challenge, researchers have employed both iterative reconstruction (IR) and deep-learning-based methods to improve image quality. For 3D PAI reconstruction, iterative methods often suffer from extremely high memory consumption and long computation time [15], [16], [17], [18], [19], [20], [21]. Even relatively advanced iterative methods [22] needs hours to reconstruct a volume (e.g., a 25.6mm×25.6mm×25.6mm region at 0.1mm resolution) of the PA data acquired by a hemispherical array system. Additionally, current artifact removal and image quality enhancement methods based on deep learning – including both supervised [23], [24], [25], [26] and unsupervised [27], [28], [29] approaches – typically require extensive datasets for network pretraining. These methods show poor transferability, which is hard to deploy for 3D PAI systems with significant system variability.

Thus, a self-supervised neural network-based method that does not require any pretraining on external datasets is desired. Reconstruction artifacts in PAI share certain similarities with noise in images—their spatial distribution and fluctuations are significantly different from true signals. To achieve zero-shot denoising (removing noise in the target image without task-specific training), a method called Zero-Shot Noise2Noise [30], which builds upon Noise2Noise [31] and Neighbor2Neighbor [32], generates two down-sampled images from noisy input data through fixed filtering and optimizes the network using residual and consistency losses, enabling the network to learn noise patterns from the noisy images themselves.

Inspired by Zero-Shot Noise2Noise, we propose Zero-Shot Artifact2Artifact (ZS-A2A), a zero-shot self-supervised artifact removal method based on a super-lightweight network. Unlike Zero-Shot Noise2Noise, ZS-A2A is tailored to the property of reconstruction artifacts in PAI. It generates pairs of downsampled reconstruction results by applying randomized dropping in the input data and further optimizes the network with residual and consistency losses. This enables the neural network to learn the artifact pattern within the input image data.

ZS-A2A does not require any pretraining on external datasets or any prior knowledge about the artifacts. It can complete artifact learning and inference for a 256 × 256 input slice in about 8 s, and for a 256 × 256 × 256 volume of 3D PAI in about 25 min. We validated the artifact removal capability of ZS-A2A on both simulated data and real invivo animal studies.

## 2 Methods

It is well-known that reconstruction artifacts arise and become more pronounced as the degree of sparsity in the detection surface increase, eventually overwhelming the reconstructed true signal. In general, when altering the number of array elements, true PA signals and artifacts in reconstructed images exhibit distinctly different fluctuation characteristics, and the reconstruction artifacts are sensitive to randomized data loss.

Based on this fact, we developed a lightweight neural network to establish a zero-shot self-supervised framework, Zero-Shot Artifact2Artifact (ZS-A2A), to remove artifacts from PA reconstruction results and enhance image quality.

**Fig. 1:**
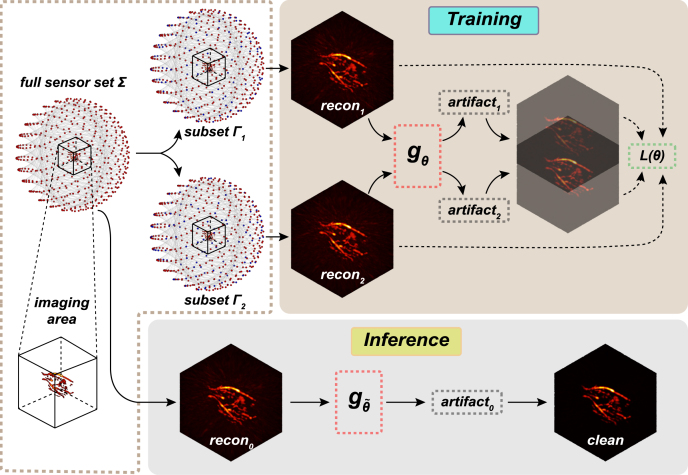
The pipeline of the Zero-Shot Artifact2Artifact.

The workflow of ZS-A2A is illustrated in Fig. 1. Specifically, for a 3D PAI system with a total of N detectors, the original PA data Σ consists all detectors, then we randomly discarding detectors to form a subset consisting of M detectors (Eq. (1)).

(1) $$\Gamma_{j}=\text{\{}n_{1}^{j},n_{2}^{j},\ldots ,n_{M}^{j}|n_{i}^{j}\in \left\{1,2,\ldots ,N\right\},$$

$$n_{i}\text{is selected after randomly discarding, and}M\leq N\text{\}},$$

where $\Gamma_{j}$ represents the j-th subset of randomly selected data.

Through this randomly discarding strategy, we generated two data subsets, $\Gamma_{1}$ and $\Gamma_{2}$, which then independently reconstructs two results ${\mathrm{recon}}_{1}$ and ${\mathrm{recon}}_{2}$ using the UBP method, respectively. These two reconstruction results are then paired and fed into LatentSpace-ArtifactPredictor, a lightweight convolutional neural network [30] $g_{\theta }$, which converts the input photoacoustic image with artifacts into a latent space representation and then predicts the artifacts as the output of the network: ${\mathrm{artifact}}_{1}=g_{\theta }\left({\mathrm{recon}}_{1}\right)$ and ${\mathrm{artifact}}_{2}=g_{\theta }\left({\mathrm{recon}}_{2}\right)$. The network parameters $\overset{˜}{\theta }$ were optimized by minimizing the loss function L(θ). Subsequently, we use the optimized network to predict the ${\mathrm{artifact}}_{0}$ from the UBP reconstruction result ${\mathrm{recon}}_{0}$ of the original PA data Σ. Finally, by subtracting the predicted ${\mathrm{artifact}}_{0}$ from ${\mathrm{recon}}_{0}$, the clean reconstruction result can be obtained: $\mathrm{clean}={\mathrm{recon}}_{0}-g_{\overset{˜}{\theta }}\left({\mathrm{recon}}_{0}\right)$.

For ZS-A2A, the loss function L(θ) is composed of a residual loss $L_{\mathrm{res.}}\left(\theta \right)$ and a consistency loss $L_{\mathrm{cons.}}\left(\theta \right)$. The residual loss uses a symmetric loss form [30], [33], as follows,

(2) $$L_{\text{res.}}\left(\theta \right)=\frac{1}{2}({\left\|{\mathrm{recon}}_{1}-g_{\theta }\left({\mathrm{recon}}_{1}\right)-{\mathrm{recon}}_{2}\right\|}_{2}^{2}$$

$$\;+{\left\|{\mathrm{recon}}_{2}-g_{\theta }\left({\mathrm{recon}}_{2}\right)-{\mathrm{recon}}_{1}\right\|}_{2}^{2}).$$

**Fig. 2:**
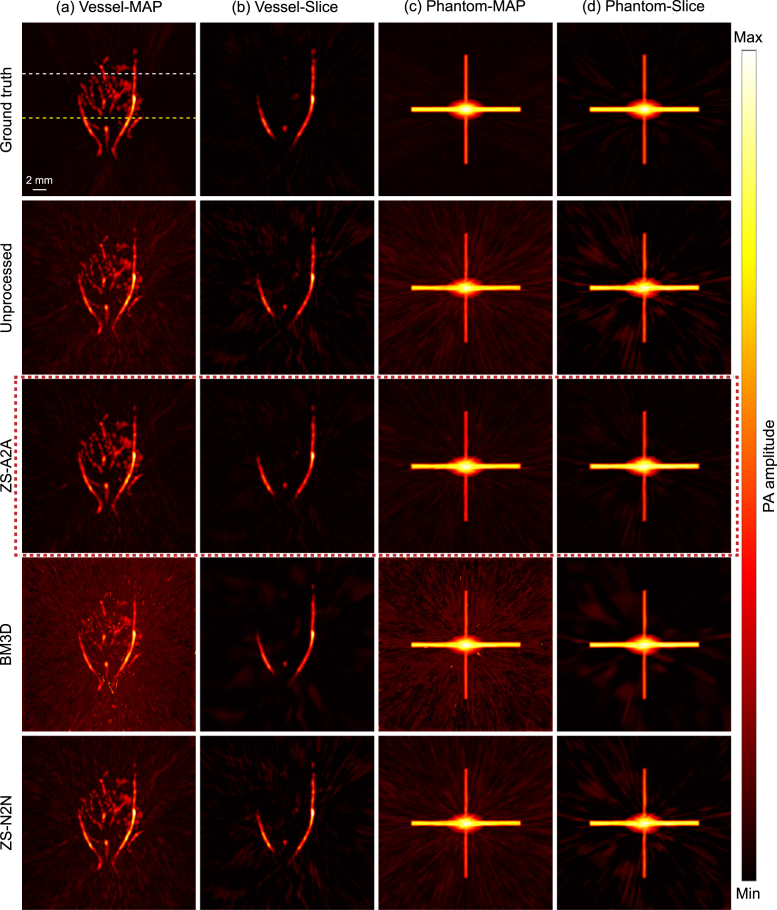
Comparison of results using different zero-shot artifact removal methods. (a) Artifact removal results for image of complex vessel-MAP. (b) Artifact removal results for images of complex vessel-slice. (c) Artifact removal results for image of simple phantom-MAP. (d) Artifact removal results for images of simple phantom-slice. (Scale: 2.0 mm.).

**Fig. 3:**
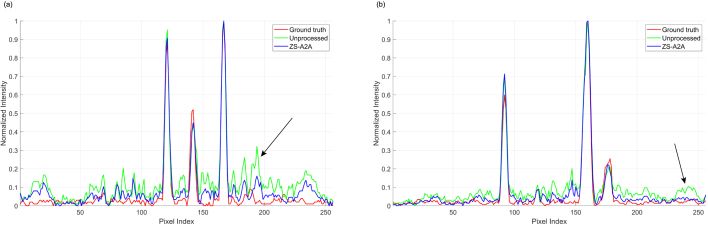
Comparison of PA intensity in vessel-MAP before and after artifact removal using ZS-A2A. (a) Intensity distribution curves along the white dashed line in the vertical coordinate of the vessel-MAP. (b) Intensity distribution curves along the yellow dashed line in the vertical coordinate of the vessel-MAP. (For interpretation of the references to color in this figure legend, the reader is referred to the web version of this article.)

Additionally, considering that reconstruction results ${\mathrm{recon}}_{1}$ and ${\mathrm{recon}}_{2}$ should converge toward the same high-quality PAI reconstruction result after artifact removal, we impose a consistency loss to serve as a regularization constraint. The consistency loss ensures that the predicted artifact-corrected reconstructions are aligned, encouraging stability and coherence in the reconstruction process. It can be formulated as:

(3) $$L_{\text{cons.}}\left(\theta \right)=\|\left({\mathrm{recon}}_{1}-g_{\theta }\left({\mathrm{recon}}_{1}\right)\right)-{\left({\mathrm{recon}}_{2}-g_{\theta }\left({\mathrm{recon}}_{2}\right)\right)\|}_{2}^{2}.$$

Therefore, the total loss function is $L\left(\theta \right)=L_{\mathrm{res.}}\left(\theta \right)+L_{\mathrm{cons.}}\left(\theta \right)$, and through gradient descent to minimize L(θ), we get the network parameters $\overset{˜}{\theta }$ for ZS-A2A.

## 3 Experiments and results

### 3.1 Simulation validation of artifact removal for PAI

We first validated ZS-A2A in simulation studies. The simulated detector array was configured as a spherical array with a radius of 60 mm, containing 2,048 detector elements arranged uniformly across the entire sphere surface. We used both simple phantom and complex vessel as the simulated PA source, which were located within a cubic region of $x,y,z\in \left(-12.8\text{mm},12.8\text{mm}\right)$ inside the spherical array (assuming the origin of the coordinate system is located at the center of the spherical array).

We used reconstruction results from a full set of 2,048 detectors as the ground truth and uniformly down-sampled 512 detectors to simulate PAI reconstruction under sparse detector configurations, resulting in artifact-contaminated results to be processed. Subsequently, we applied a random discarding strategy to generate subsets of data. For both simple phantom and the complex vessel, the subset contained 400 detectors. The reconstruction grid resolution was set to 0.1 mm.

In the simulation study, the reconstructed 3D image was first divided into a series of two-dimensional slices, and we applied ZS-A2A on each slice for artifact removal. We also compared with Zero-Shot Noise2Noise (ZS-N2N), another zero-shot method, as well as the classical BM3D [34] algorithm. To ensure fairness, the results of all methods were compared based on processing the volume slice by slice. The comparative results are shown in Fig. 2. To further evaluate the artifact removal performance, metrics including Peak Signal-to-Noise Ratio (PSNR) and structural similarity index measure (SSIM) were calculated for both slice and the maximum amplitude projection (MAP) of the reconstructed volume, as displayed in Table 1, Table 2. It is evident that ZS-A2A achieved prominently better performance across nearly all tasks.

For the artifact removal of slices from the complex vascular structure, ZS-A2A improved the PSNR by more than 6 dB compared to the unprocessed image ( Table 1). This is a significant enhancement for images with an original PSNR around 30 dB. Additionally, from the perspective of SSIM (as shown in Table 2), ZS-A2A demonstrates an unparalleled ability to enhance image quality. This improvement is also evident in the results displayed in Fig. 2. We further reconstructed values along the white and yellow dashed lines in the MAP (Fig. 2(a)) to validate the numerical accuracy before and after applying ZS-A2A (Fig. 3). It can be observed that after using ZS-A2A, the intensity of the artifacts significantly decreases (as indicated by the black arrows), while the true signals remain largely unchanged. The artifact removal effectiveness for the complex vascular structure signals the potential of ZS-A2A to significantly enhance the quality of real PA images.

**Fig. 4:**
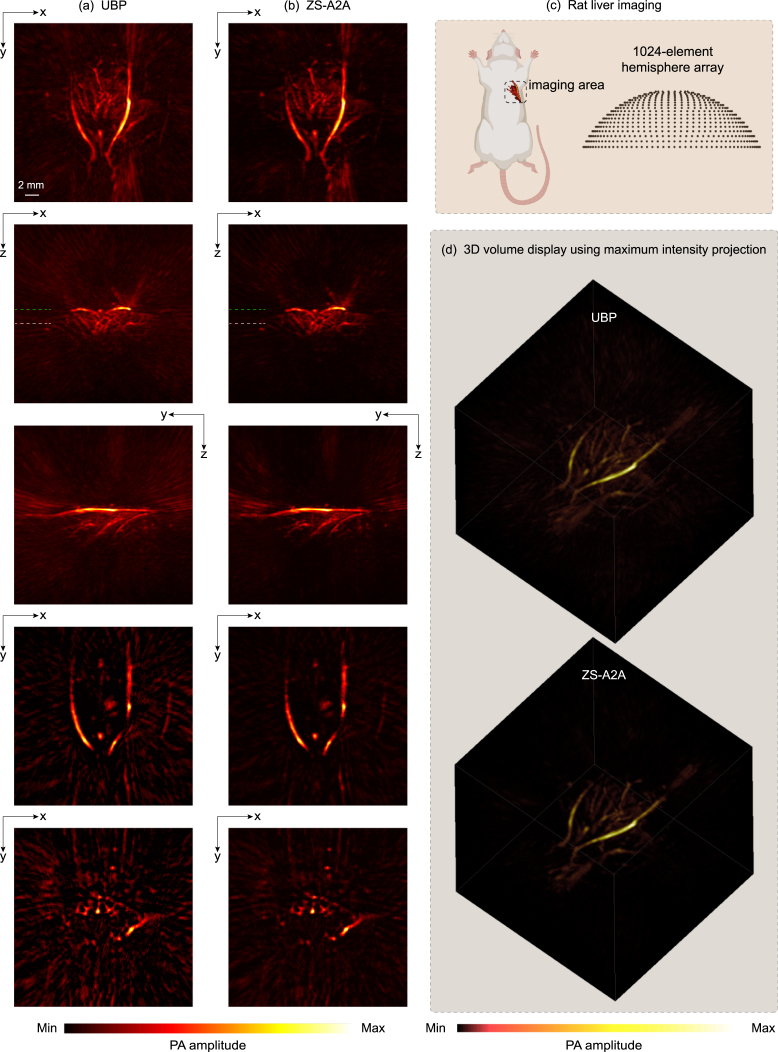
3D PA reconstruction results of a rat liver. (a) XY Plane-MAP, XZ Plane-MAP, YZ Plane-MAP and the cross-section slice at green dashed line and white dashed line marked in XZ Plane-MAP of the UBP 3D reconstruction results using 1,024 sensor signals. (b) XY Plane-MAP, XZ Plane-MAP, YZ Plane-MAP and the cross-section slice at green dashed line and white dashed line marked in XZ Plane-MAP of the UBP 3D reconstruction results after ZS-A2A artifact removal using 1,024 sensor signals. (Scale: 2 mm.) (c) Schematic diagram of the imaging area. (d) 3D volume display of the reconstruction results using maximum intensity projection. (For interpretation of the references to color in this figure legend, the reader is referred to the web version of this article.)

**Table 1:** PSNR (in dB) of simulated PAI image results using different zero-shot artifact removal methods in Eq. (2).

Image	Vessel-MAP	Vessel-Slice	Phantom-MAP	Phantom-Slice
Unprocessed	27.64	34.79	26.26	32.36
ZS-A2A	**33.99**	**38.81**	**32.84**	**35.62**
BM3D	21.36	37.31	23.32	33.91
ZS-N2N	27.79	34.81	26.65	32.85

**Table 2:** SSIM (in percentage) of simulated PAI image results using different zero-shot artifact removal methods in Fig. 2.

Image	Vessel-MAP	Vessel-Slice	Phantom-MAP	Phantom-Slice
Unprocessed	68.28	72.07	67.62	68.32
ZS-A2A	**86.97**	**78.31**	**86.83**	**75.03**
BM3D	38.90	78.26	44.99	72.79
ZS-N2N	69.06	73.55	69.08	70.14

### 3.2 Artifact removal for in vivo animal studies

Then, we validated ZS-A2A for the 3D PAI reconstruction results of invivo rat experimental data. The invivo animal study data, including rat liver and rat kidney, was acquired by Kim’s lab using a hemispherical ultrasound (US) transducer array with 1,024 elements and a radius of 60 mm [35]. Each detector in the array has a center frequency of 2.02 MHz and a bandwidth of 54%. The effective field of view (FOV) was 12.8mm×12.8mm×12.8mm, and the spatial resolutions of approximately 380 μ m were nearly isotropic in all directions when all 1,024 US transducer elements were used [35]. More details regarding the 3D PAI system and animal experiments can be found in literatures [35], [36], [37].

We generated subsets of data using a random discarding strategy, retaining 800 detectors out of 1,024 total detectors for our experiments. For the 3D PAI reconstruction results, we applied ZS-A2A to each slice independently, without any modifications to the network’s training parameters.

**Fig. 5:**
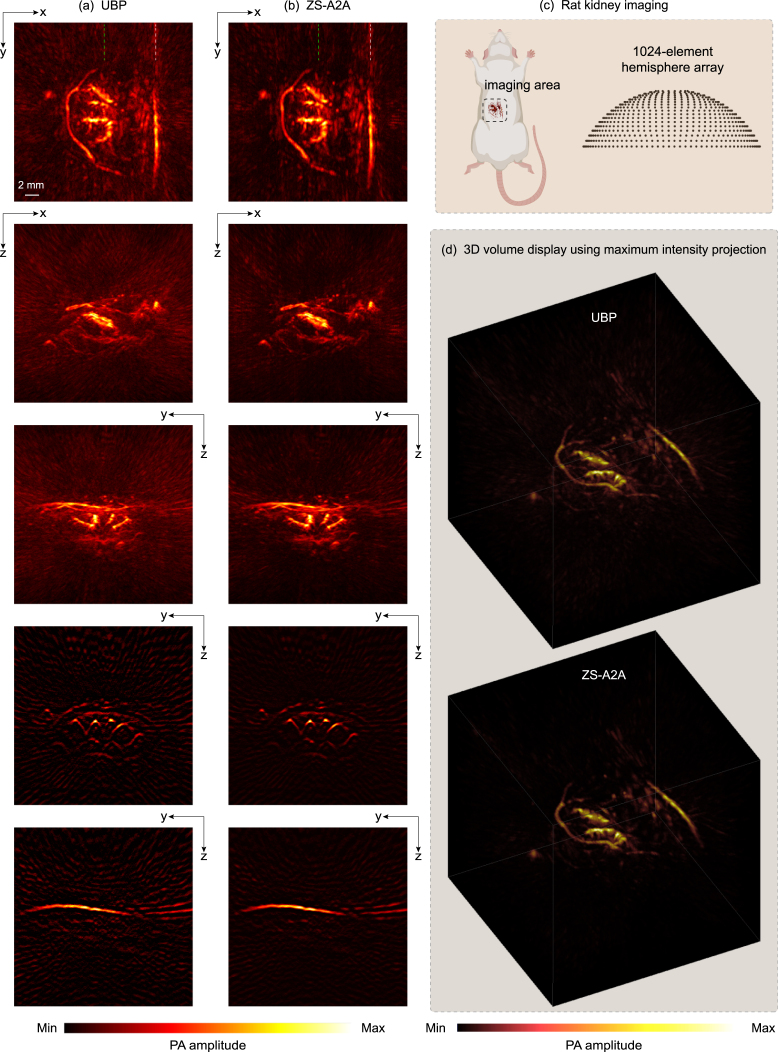
3D PA reconstruction results of a rat kidney. (a) XY Plane-MAP, XZ Plane-MAP, YZ Plane-MAP and the cross-section slice at green dashed line and white dashed line marked in XY Plane-MAP of the UBP 3D reconstruction results using 1,024 sensor signals. (b) XY Plane-MAP, XZ Plane-MAP, YZ Plane-MAP and the cross-section slice at green dashed line and white dashed line marked in XY Plane-MAP of the UBP 3D reconstruction results after ZS-A2A artifact removal using 1,024 sensor signals. (Scale: 2 mm.) (c) Schematic diagram of the imaging area. (d) 3D volume display of the reconstruction results using maximum intensity projection. (For interpretation of the references to color in this figure legend, the reader is referred to the web version of this article.)

We presented the reconstruction results of a rat liver and rat kidney in Fig. 4, Fig. 5, respectively. Fig. 4(a-b) and Fig. 5(a-b) (from top to bottom) show the maximum amplitude projection (MAP) images along three orthogonal directions of the original 3D reconstruction results obtained using UBP and those after applying ZS-A2A. The slices corresponding to the green and white dashed lines are also displayed. The results clearly demonstrate ZS-A2A’s superior artifact removal performance.

We further compared the Contrast-to-Noise Ratio (CNR) of slice results before and after applying ZS-A2A. For the liver, the CNR improved from 17.48 to 43.46, and for the kidney, the CNR increased from 21.56 to 46.31. In addition, we visualized the 3D results (based on maximum intensity projection) before and after artifact removal in Fig. 4(d) and Fig. 5(d). The visualizations show a significant enhancement in reconstruction quality, further confirming the effectiveness of ZS-A2A.

**Fig. 6:**
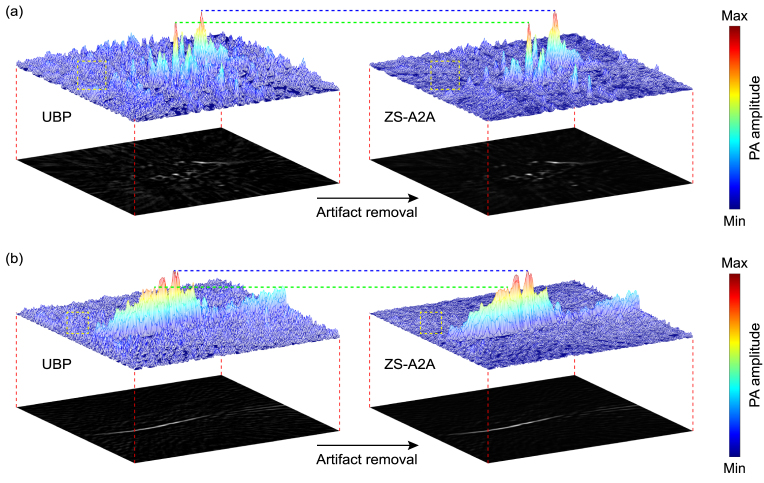
Comparison of PA amplitude in reconstruction results before and after artifact removal using ZS-A2A. (a) 3D distribution of PA amplitude for the slice along the white dashed line in the XZ Plane-MAP of the rat liver. (b) 3D distribution of PA amplitude for the slice along the white dashed line in the XY Plane-MAP of the rat kidney.

To demonstrate the accuracy of ZS-A2A in removing artifacts while preserving the true PA signals almost unchanged, we present the 3D distributions of PA amplitude values before and after artifact removal for the rat liver (Fig. 6(a)) and kidney (Fig. 6(b)). In the figure, the blue and green dashed lines are used to highlight and compare the PA amplitudes of characteristic true PA signals, showing that the actual PA signal amplitudes remain high accuracy after applying ZS-A2A. In contrast, the artifact regions, marked by yellow dashed boxes, display significant changes with notably reduced amplitudes, clearly illustrating the effectiveness of ZS-A2A in artifact removal.

### 3.3 Computational time

Computation time is an essential metric for evaluating the efficiency of a model. All the experiments were conducted on a single NVIDIA GeForce RTX 3090 Ti. In our experiments, the number of iterations was set to 3,000, with a learning rate of 0.01, a learning rate decay schedule of step_size=1,000, and γ=0.6. For a single slice with the size of 256 × 256, corresponding to the reconstructed region of a typical PA hemispherical array imaging system (25.6mm×25.6mm×25.6mm) with a reconstruction grid resolution of 0.1 mm, ZS-A2A took less than 8 s to complete both the learning and artifact removal inference, and for the entire 3D reconstruction (as in the invivo experimental data validation presented in this work), the process took only 25 min to complete the entire learning and inference for artifact removal.

### 3.4 Ablation studies

In ZS-A2A, only two subsets of data via randomly discarding are used to learn the artifact patterns. Interestingly, when the number of subsets is increased, the artifact removal performance of the model becomes worse. For instance, when we increased the number of subsets to 3 and correspondingly modified the calculation of residual and consistency losses for a simulated complex vessel image. On the same NVIDIA GeForce RTX 3090 Ti, we observed that, compared to using 2 subsets, the computation time increased from 8 s to 11 s, while the PSNR dropped from 38.81 to 38.29. When the number of subsets was further increased to 4, the computation time rose to 16 s, and the PSNR remained approximately 38.28. This is likely because the network begins to learn deeper patterns of artifacts under the randomized perturbations, which however does not benefit more to distinguish these artifacts from the true PA signals. This demonstrates that 2 subsets provides the optimal artifact removal performance for the ZS-A2A framework in our study cases.

Additionally, like ZS-N2N, ZS-A2A employs a very simple two-layer image-to-image network [30]. The network consists of two convolution operators with kernel sizes of 3 × 3, followed by a 1 × 1 convolution operator, resulting in a total of approximately 22k parameters. Unlike ZS-N2N’s findings, where increasing network complexity significantly degraded performance (e.g., using U-Net for ZS-N2N led to notable reductions in denoising efficacy [30]), our study revealed that a slight increase in network complexity does not significantly impair artifact removal performance for ZS-A2A. Specifically, we experimented with a relatively shallow U-Net [38] (only two down-sampling/up-sampling layers) with approximately 168k parameters. Across multiple tests, the PSNR fluctuated around 38.52, comparable to the lightweight convolutional network’s result of 38.81. However, it is important to note that, for a image size of 256 × 256, the shallow U-Net required approximately 41 s for learning and inference on the same NVIDIA GeForce RTX 3090 Ti, which is significantly slower than the lightweight convolutional network (approximately 8 s). Therefore, although the shallow U-Net achieves comparable artifact removal performance, its efficiency is far inferior. These results suggest that the current lightweight convolutional network structure used in ZS-A2A is both highly effective and efficient, making it particularly suitable for practical applications.

## 4 Discussions

In this study, we proposed a method that leverages the unique properties of PAI artifacts and introduces a novel self-supervised learning paradigm that obviates the need for extensive training datasets or prior knowledge of the artifacts. The performance of ZS-A2A has been evaluated through both simulation studies and invivo animal experiments, demonstrating its superior artifact removal capabilities compared to existing zero-shot methods such as Zero-Shot Noise2Noise (ZS-N2N) and classical BM3D algorithms. In the simulation studies, ZS-A2A achieved very promising performance results across various metrics, including Peak Signal-to-Noise Ratio (PSNR) and structural similarity index measure (SSIM). In the invivo animal studies, ZS-A2A demonstrated its robustness and versatility by effectively removing artifacts from 3D PAI reconstructions of rat liver and kidney data. The quantitative improvements in CNR, from 17.48 to 43.46 for the liver and from 21.56 to 46.31 for the kidney, further validated the method’s efficacy. From a computational perspective, ZS-A2A’s lightweight network architecture ensures rapid convergence and inference times, making it highly suitable for real-time applications. On a single NVIDIA GeForce RTX 3090 Ti GPU, ZS-A2A could process a single 256 × 256 slice in less than 8 s and a full 3D volume in approximately 25 min. This computational efficiency is a critical advantage over iterative methods, which often require hours to process similar datasets.

However, ZS-A2A still has certain limitations. When the detector array is overly sparse or the angular coverage is severely restricted, ZS-A2A may not be able to effectively eliminate artifacts. Additionally, under the same detector data conditions, the reconstruction quality of ZS-A2A cannot match that achieved by iterative methods. Nevertheless, considering computational efficiency, ZS-A2A demonstrates greater practicality and usability compared to iterative methods.

In conclusion, the development of ZS-A2A represents a significant step forward in improving the quality of PAI reconstructions. Its zero-shot learning capability, combined with computational efficiency and robust performance, makes it a highly promising tool for enhancing the clinical utility of PAI. Furthermore, the ZS-A2A method has the potential to be extended to other imaging modalities such as positron emission tomography (PET) and single photon emission computed tomography (SPECT), offering a generalized framework for artifact removal in medical image reconstruction. It may serve as a universal solution for enhancing the quality of reconstructed images across a wide range of medical imaging techniques.

## CRediT authorship contribution statement

**Shuang Li:** Writing – review & editing, Writing – original draft, Visualization, Validation, Software, Resources, Project administration, Methodology, Investigation, Formal analysis, Data curation, Conceptualization. **Qian Chen:** Methodology, Conceptualization. **Chulhong Kim:** Resources, Funding acquisition, Data curation. **Seongwook Choi:** Resources, Data curation. **Yibing Wang:** Resources. **Yu Zhang:** Resources. **Changhui Li:** Writing – review & editing, Supervision, Funding acquisition.

## Declaration of competing interest

The authors declare that they have no known competing financial interests or personal relationships that could have appeared to influence the work reported in this paper.

## Data Availability

The source codes and data for ZS-A2A are available in the following GitHub repository: https://github.com/JaegerCQ/ZS-A2A.
